# Ability of *Spalangia endius* (Hymenoptera: Pteromalidae) to Parasitize *Bactrocera dorsalis* (Diptera: Tephritidae) after Switching Hosts

**DOI:** 10.3390/insects12070613

**Published:** 2021-07-07

**Authors:** Yuan Zheng, Zi-Wei Song, Yu-Ping Zhang, Dun-Song Li

**Affiliations:** 1 College of Plant Protection, South China Agricultural University, Guangzhou 510642, China; zhengyuan506@163.com; 2 Guangdong Provincial Key Laboratory of High Technology for Plant Protection/Plant Protection Research Institute, Guangdong Academy of Agricultural Sciences, Guangzhou 510640, China; ziweisong@139.com (Z.-W.S.); zhangyp@gdppri.cn (Y.-P.Z.)

**Keywords:** parasitoids, functional response, switching host, biological control, parasitoid age

## Abstract

**Simple Summary:**

*Bactocera dorsalis* is an insect pest that causes substantial losses to fruit crops. It can be potentially controlled by the parasitoid wasp, *Spalangia* *endius* Walker (Hymenoptera: Pteromalidae). *S. endius* is also used to control *Musca domestica* and is commercially produced. We studied the parasitism capacity of *S. endius* as a pupal parasite of *Bactocera dorsalis* after switching hosts. We mass-reared *S. endius* for more than 50 generations on *M. domestica*, and then allowed them to parasitize *B. dorsalis* to study the parasitism capacity of *S. endius*. More *M. domestica* were parasitized than *B. dorsalis* at different host densities. The *S. endius* colony, which was reared on *M. domestica* can be used to control *B. dorsalis* at a low density of *B. dorsalis.* The parasitism capacity of *S. endius* could be improved. The result showed that parasitoid-pest ratio should be 1:25 in order to maintain a relatively stable parasitism rate for controlling *B. dorsalis.* The rate of *S. endius* parasitizing *B. dorsalis* was decreased by parasitoid age. These results will help to optimize the use of *S. endius*, reared on *M. domestica*, for control of *B. dorsalis*.

**Abstract:**

We studied the parasitism capacity of *Spalangia endius* as a pupal parasite of *Bactocera dorsalis* after switching hosts. We used pupae of *B. dorsalis* and *M. domestica* as the hosts and studied parasitism by *S. endius* in the laboratory. The parasitism capacities were compared at different host densities and different parasitoid ages. The two functional responses of *S. endius* fitted a Holling Type II equation. More *M. domestica* were parasitized than *B. dorsalis* at all the densities. The ability of *S. endius* to control *M. domestica* was *α*/*T*_h_ (parasitism capacity) = 32.1950, which was much stronger than that of control *B. dorsalis*, which was *α*/*T*_h_ = 4.7380. The parasitism rate of wasps that had parasitized *B. dorsalis* had decreased by the emergence time of parasitoids. These results suggest that the parasitoid-pest ratio should be 1:25 to maintain a relatively stable parasitism rate for control of *B. dorsalis.* The *S. endius* colony reared on *M. domestica* successfully controlled a low-density population of *B. dorsalis* in the lab. We provide evidence suggesting that the parasitism capacity of *S. endius* needs to be improved.

## 1. Introduction

*Bactrocera dorsalis* Hendel (Diptera: Tephritidae) is a worldwide pest of fruit, causing great economic loss [1,2]. This species is mainly controlled by the use of insecticides. However, insecticides are often ineffective because females oviposit, and larvae develop, within fruits where they are protected. Insecticide application as a primary method for *B. dorsalis* control is also compromised by insecticide resistance [3], and insecticides have detrimental effects on humans, animals, and the environment [4]. Male annihilation, attractant sprays, and bagging have been studied as components of integrated management of *B. dorsalis* and other fruit flies, but male annihilation and attractant sprays were not satisfactory, they can only be used to trap for killing males [5], and fruit bagging is expensive and labor-intensive, usually used for large fruits or fruits with high economic value [6]. Simple and effective strategies for *B. dorsalis* control are needed.

Biological control helps reduce *B. dorsalis* populations and is more environmentally friendly than many other methods [7]. Many wasp species parasitize *B. dorsalis* eggs or larvae. Good control has previously been demonstrated using parasitic wasps to control *B. dorsalis*. *Fopius arisanus* (Sonan), *Diachasmimorpha longicaudata* (Ashmead), *Fopius vandenboschi* (Fullaway)*,* and *Psyttalia incisi* (Silvestri) have been used to control *B. dorsalis* in Hawaii from 1951 to the present [8,9,10,11]. *Biosteres arisanus* (Sonan) was effective at colonizing fruit fly hosts when fly densities and fruit abundance were relatively low in guava orchards [12]. *D. longicaudata* was introduced in Taiwan in 1985 and was an effective parasitoid [11]. However, there are few wasp species that can parasitize *B. dorsalis* pupae. Hence, it is important to develop the use of a new parasitoid in the pupal stage to control *B. dorsalis*.

*Spalangia endius* Walker is a pupal parasitoid of several dipteran species. These include *B. dorsalis*, *Bactrocera correcta* (Bezzi), *Zaprionus indianus* Gupta, and *Bactrocera cucurbitae* [7,13,14,15]. *S.* *endius* was reared on *B. dorsalis* for several generations and retained parasitic ability on *B. dorsalis* [16]. Zhang et al. [17,18] indicated that each parasitoid could parasitize 31.55 pupae of *B. dorsalis* within 24 h at 26 °C, RH 70%, h L:D = 14:10, and the longevity of *S. endius* was about 10.3 d when reared on *B. dorsalis*. *S. endius* can, therefore, be used for the biocontrol of *B. dorsalis*. *B. dorsalis* larvae are reared on a banana and maize-based artificial diet [19]. These take much time to prepare the artificial diet, and will increase the cost of mass production of *S. endius*. Therefore, we choose to rear *S. endius* on *Musca* *domestica*.

*Musca domestica* L. (Diptera: Muscidae) is an important pest of animal and agriculture industries. It causes medical risks for humans by spreading many diseases near livestock farms [20]. There are many commercially available filth fly parasitoids used against *M. domestica*. *S. endius* is commonly used against *M. domestica* in the USA [21]. *M. domestica* larvae are fed on a wet wheat-based artificial diet, it is more easily commercially reared on artificial diets than *B.* *dorsalis*, and *S.* *endius* can be reared on *M. domestica*. We conducted mass rearing of *S. endius* using *M. domestica*, then, we used the parasitoid to control *B.* *dorsalis*. However, the parasitism rate may decrease after continuous mass rearing of parasitoids on a substitutive host. For example, the searching and parasitism of *Scleroderma sichuanensis* Xiao decreased when *S. sichuanensis* was reared, long term, on *Tenebrio molitor* L. as a substitutive host [22]. Therefore, we studied the parasitism rate to estimate the parasitism capacity of *S. endius* after switching hosts to confirm whether *M. domestica* was a suitable host to rear *S. endius* for controlling *B. dorsalis.*

In addition, host density is reported to affect the performance of a parasitoid [23]. The functional response of a parasitoid can be inversely or positively host-density-dependent, or independent from host density [24]. Type Ι functional responses result in density-independent host parasitizing, type II in a negatively density-dependent response where with increasing host density a decreasing percentage of host is parasitized, and type III in a positively density-dependent response, where, over a certain range of increasing host densities, an increasing percentage of host is parasitized [25]. The type of functional response is an essential factor in the selection of efficient biological control agents, how a parasitoid responds to an increasing host population can determine the success of biological control [26]. Parasitism is usually affected by parasitoid age. For instance, two days old parasitoid *Telenomus remus* parasitized the maximum number of eggs in the different hosts, i.e., *Corcyra cephalonica*, *Helicoverpa armigera*, *Spodoptera litura*, and *S. exigua* [27]. 1-day-old *Trichogramma cacoeciae* Marchal produced fewer parasitized eggs than 2, 3, and 4-day-old females when parasitized *Lobesia botrana* Denis [28]. The optimum age for *Ooencyrtus mirus* parasitized on *Bagrada hilaris* (Burmeister) was 3–10 d [29]. The parasitism rate was higher in older (5 to 10 d) than younger (1 to 4 d) adults of *Telenomus podisi* when parasitizing *Euschistus heros* (Fabricius) or *Dichelops melacanthus* (Dallas) [30]. In this paper, we described the results of the functional response of *S. endius* on two hosts at different densities and the parasitism of *S. endius* on *B. dorsalis* at different parasitoid ages. The data will help determine how to use *S. endius*, mass-reared on *M. domestica*, as a biocontrol agent to manage *B. dorsalis* populations.

## 2. Materials and Methods

Flies and Parasitoids—The *B. dorsalis* strain originated from a population collected at Guangzhou, China. About 600 flies were housed in a screen cage (50 × 50 × 30 cm) and supplied with water, sugar, and yeast extract. Larvae were reared on a banana and maize-based artificial diet [19].

The *M. domestica* strain used had been reared in the laboratory for five years. Adults were housed in a screen cage (50 × 50 × 30 cm) and supplied with water, sugar, and milk powder. Larvae were fed on a wet wheat-based artificial diet in plastic containers (57 ×37 × 9 cm).

A laboratory population of *S. endius* was primarily obtained from *B. dorsalis* infesting guava in Guangzhou. The *S. endius* colony was maintained on *M. domestica* pupae up to 50 generations (more than 3 years). The *S. endius* colony (about 100 pairs of zero-to-10-d-old) was placed inside the oviposition cages (50 × 50 × 30 cm) provided with water and honey.

All experiments were conducted under the same laboratory conditions controlled at 26 °C ± 2 °C under a 14:10 h (L:D) photoperiod and 70% ± 10% relative humidity (RH).

Functional response experiment—In this experiment, according to the parasitic number of parasitoids in the pre-experiment, 5, 10, 15, 20, 25, or 30 one- to two-d-old *B. dorsalis* or *M. domestica* pupae were placed in plastic cups (7 cm diam. × 10 cm high). One 24-h-old mated female parasitoid was placed in each cup for 24 h and provided with cotton wool saturated with 10% honey water as food. After exposure to the parasitoid, the pupae from each treatment were covered with humid sand until emergence. The cup was covered with 80-mesh nylon and was examined on successive days for parasitoid emergence after 10 d. Ten replicates were set up for each host density. The number of emerging adult parasitoids was recorded. Unhatched pupae were dissected under a stereomicroscope to examine whether they were parasitized.

Effect of parasitoid age on parasitization—Based on the emergence time, wasps were divided into six age levels: 1 d old, 2 d old, 3 d old, 4 d old, 5 d old, and 6 d old (the 1-d-old wasps had emerged within one day, etc.). Twenty 1- to 2-day-old *B. dorsalis* or *M. domestica* pupae were placed in a glass tube (2 cm diam. × 10 cm high). Ten 24-h-old fertilized female parasitoids were placed into a tube with *B. dorsalis* or *M. domestica* pupae for 24 h. Then, the pupae from each tube were transferred into a cup (7 cm diam. × 10 cm high), and covered with wet sand (10% water) until emergence. They were examined on successive days for the emergence of parasitoids after 10 d. Each tube and cup was covered with an 80-mesh screen. Thirty replicates were established at each wasp age. The number of emerged adult parasitoids was recorded. The unhatched pupae were dissected under a stereomicroscope to examine whether they were parasitized.

Data analysis—The functional response experiments were analyzed in two steps [31]. First, a logistic regression of the proportion of parasitized hosts (*N*_e_) vs. the initial number of hosts (*N*_0_) was used to determine the shape of the functional response. The polynomial function was fitted as follows:

*N*_e_/*N*_0_ = exp(*P*_0_ + *P*_1_*N*_0_ + *P*_2_*N*_0_^2^ +*P*_3_*N*_0_^3^)/[1+exp(*P*_0_ + *P*_1_*N*_0_ + *P*_2_*N*_0_^2^ +*P*_3_*N*_0_^3^)] (1)

where *N*_e_ is the number of parasitized pupae, *N*_0_ is the initial number of host pupae, and *P*_0_, *P*_1_, *P*_2__,_ and *P*_3_ are the intercept, linear, quadratic, and cubic coefficients, respectively. The type of functional response was determined by fitting data to the model (1). A sign of *P*_1_ is negative, and the functional response is type II. A sign of *P*_1_ is positive and *P*_2_ is negative, and the functional response is type III [31].

For the next step of the analysis, the handling time and the attack rate coefficients of a type II response were estimated using the random parasitoid equation:

*N*_e_ = *N*_0_{1−exp[*α*(*T*_h_*N*_e_−*T*)]} (2)

where *N*_e_ is the number of hosts parasitized, *N*_0_ is the initial number of hosts, *T*_h_ is the handling time, and *T* is the total time available for the parasitoid (1 d).

The percentage of parasitism of *S. endius* was estimated based on Equation (3):

Parasitism percentage (%) = (*N*_p_/*N*_t_)*100 (3)

where *N*_p_ is the number of parasitized pupae, and *N*_t_ is the total number of host pupae in one treatment (=20 here).

The percentage of emergence of *S. endius* was estimated based on Equation (4):

Emergence percentage (%) = (*N*_e_/*N*_p_)*100 (4)

where *N*_e_ is the number of emerging parasitoids, and *N*_p_ is the number of parasitized pupae.

The data were analyzed using the SAS V9.0 software (SAS Institute Inc., Cary, NC, USA). The NLIN procedure in SAS was used to estimate the attack rate (*α*) and handling time (*T*_h_) parameters. Significant differences among different treatments were determined by one-way ANOVA followed by Tukey’s test (*p* = 0.05). The effects of parasitoid ages on parasitization were analyzed by regression analysis (SAS 2007).

## 3. Results

### 3.1. Functional Response

Based upon the logistic regression (Table 1), both linear coefficients (*P*_1_) were negative and quadratic coefficients (*P*_2_) were positive. This suggested that the functional response of *S. endius* parasitizing *B. dorsalis* or *M. domestica* was type II. The attack rate (*α*) of *B. dorsalis* was 0.6908, which was lower than that of *M. domestica* (1.0399) (Table 2). The handling time (*T*_h_) of *B. dorsalis* was 0.1458 d, which was much longer than that of *M. domestica* (0.0323 d). The ability of *S. endius* to control *M. domestica* was *α*/*T*_h_ = 32.1950, which was much stronger than the ability to control *B. dorsalis* (*α*/*T*_h_ = 4.7380). These data indicated that *S. endius* had a better effect in parasitized *M. domestica* than in *B. dorsalis*.

**Figure 1:**
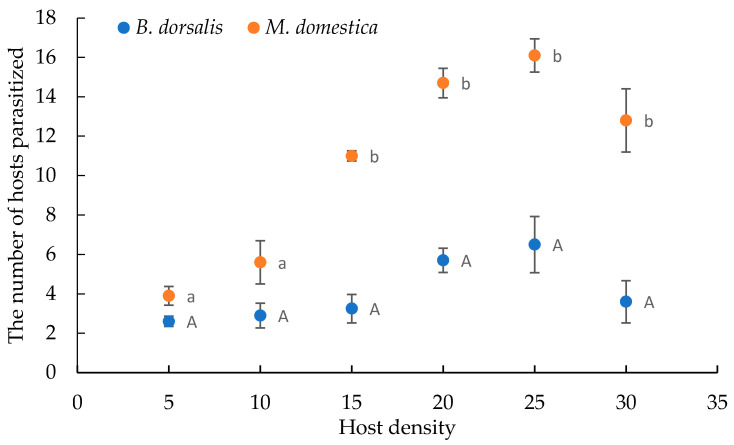
Numbers of *B. dorsalis* and *M. domestica* pupae parasitized by *S. endius* in relation to host density. Different small or capital letters are significantly different (*p* < 0.05, *t*-test).

These results showed that the numbers of *B. dorsalis* and *M. domestica* parasitized by *S. endius* increased until a certain density, and thereafter, decreased as the density increased. At the same host densities, *S. endius* parasitized more pupae of *M. domestica* than those of *B. dorsalis* (Figure 1). In the low-level host densities (5 and 10), the number *S. endius* parasitized pupae of *M. domestica* and *B. dorsalis* was not significantly different. When the host densities exceeded 15, *S. endius* parasitized significantly more pupae of *M. domestica* than of *B. dorsalis*.

### 3.2. Effect of Parasitoid Ages on Parasitization

**Figure 2:**
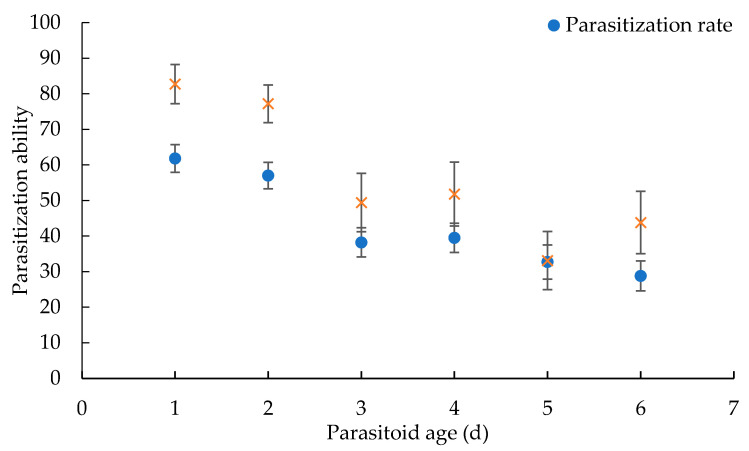
Parasitization ability of *S. endius* on *B. dorsalis* at different ages. Parasitization ability means the parasitization rate and emergence percentage of *S. endius*.

The parasitism ability of *S. endius* at different ages varied (Figure 2). The parasitism rates of wasps decreased gradually during the first 3 d after emergence (F = 34.488, *p* < 0.01). The rate of parasitism (61.8%) and emergence of wasp from parasitized *B. dorsalis* (82.7%) (F = 15.311, *p* = 0.017) were highest on the first day after emergence, and these values were significantly different from the rates on the third to the sixth day.

## 4. Discussion

The parasitism capacity of *S*. *endius* as a pupal parasite of *B*. *dorsalis* after switching hosts has been studied. Functional response studies showed that the numbers of *B. dorsalis* and *M. domestica* parasitized by *S. endius* initially increased but thereafter, decreased as the density increased (Figure 1). The results showed that the data from two treatments could fit a type II functional response curve statistically. This curve is most common for parasitoids and features a parasitism rate that decreases exponentially as host density increases [32,33]. Van Lenteren et al. [25] suggested that natural enemies with type II functional response could be used in inundative biological control. When parasitoids with type II response are applied in biological control systems, a high natural enemy to pest ratio is necessary to achieve effective pest suppression [34,35]. *S. endius* parasitized 6.5 *B. dorsalis* pupae when the host density was 25, and this was the highest number of parasitized hosts. We suggest that the parasitoid-pest ratio should be 1:25 in order to maintain a relatively stable parasitism rate in controlling *B. dorsalis*. It is necessary to determine the number of wasps before field release to achieve the best control efficiency.

The *S. endius* colony in this study was maintained on pupae of *M. domestica*, and then used to parasitize *B. dorsalis*. In the low-level host densities (5 and 10), the number *S. endius* parasitized pupae of *M. domestica* and *B. dorsalis* was not significantly different (Figure 1). We suggest that when the *B. dorsalis* density is low, the parasitoid-pest ratio should be greater than 1:10, we can use *S. endius* reared on *M. domestica* to control *B. dorsalis*. However, the use of the same hosts to rear parasitoids for successive generations under a constant temperature may result in degeneration of the wasps and reduced ability to attack the target host [36]. These problems are well-known in artificial breeding of insects, for example, the breeding effect decreased after several generations in rearing *Sclerodema guani* Xiao by using the pupae of *Antheraea pemyi* [37]. The main reasons include degeneration of genetic factors. Moreover, some insect species require specific environmental conditions to maintain their health [38]. The attack rate of *S. endius* parasitizing *B. dorsalis* was estimated to be 0.6908 ± 0.5692. It was similar to that of *Spalangia longepetiolata* (Boucek), which was 0.6526 [39]. The handling time of *S. endius* parasitized *B. dorsalis* was estimated to be 0.1458 ± 0.0657 d, which was longer than that of *S. longepetiolata* (0.0571 d) [39]. The results showed more *M. domestica* were parasitized than *B. dorsalis* at all densities. The ability of *S. endius* to control *B. dorsalis* was *α*/*T*_h_ = 4.7380, which was much less than that of the ability to control *M. domestica*, which was *α*/*T*_h_ = 32.1950. These results suggested that *M. domestica* is an ideal substitute host for mass rearing *S. endius.* Meanwhile, the parasitism capacity of *S. endius* could be improved using population rejuvenation techniques for the reproduction of *S. endius* in *B. dorsalis*, such as nutritional changes, crossbreeding with wild populations, or collecting the host in the field to revitalize the wasps.

In this study, the parasitism rates of wasps parasitizing *B. dorsalis* decreased gradually after emergence, from 61.8% to 28.8%, which was less than the rates reported by Zheng et al. [16]. The rate of parasitism and emergence of wasps from parasitized *B. dorsalis* reached their highest levels on the first day after emergence, and then decreased. This may be related to the timing of ovarian development. *S. endius* belonged to synovigenic insect [40]. It is speculated that the female had to feed complementary nutrition for spawning and survival, which affects their parasitism and emergence rate. Furthermore, host feeding would affect egg maturation. For example, *Diglyphus isaea* matured more eggs when they were fed on the third instar larvae of their hosts *Liriomyza sativae*, much more than that of this parasitoid fed on a carbohydrate diet, on day 5 after emergence [41]. The effects of supplementary food and host feeding on the parasitism rate require further study.

The performance of parasitoids in parasitizing hosts can be influenced by many factors. In addition to the influence of host density and parasitoid age, the temperature, humidity, and behavior characteristics should also be considered.

## 5. Conclusions

The *S. endius* colony maintained on *M. domestica* can be used to control *B. dorsalis* at a low density of *B. dorsalis*. *M. domestica* is an ideal substitute host for mass rearing *S. endius.* The parasitism rate of wasps that parasitized *B. dorsalis* decreased with emergence time of wasps. The parasitism capacity of *S. endius* could be improved for controlling *B. dorsalis*. These results will help optimize the use of *S. endius,* reared on *M. domestica,* for control of fruit flies.

## Figures and Tables

**Table 1:** Maximum likelihood analysis of the functional response for the proportion of different hosts parasitized by *S. endius*.

Host Species	Parameters	Estimate (±SE)	χ2	*p*
*B. dorsalis*	*P*_0_	2.9038 ± 0.9703	8.96	0.0028
	*P*_1_	−0.7547 ± 0.2003	14.19	0.0002
	*P*_2_	0.0448 ± 0.0121	13.67	0.0002
	*P*_3_	−0.00084 ± 0.000221	14.30	0.0002
*M. domestica*	*P*_0_	2.3848 ± 0.8892	7.19	0.0073
	*P*_1_	−0.4192 ± 0.1733	5.85	0.0156
	*P*_2_	0.0308 ± 0.0101	9.32	0.0023
	*P*_3_	−0.00067 ± 0.000179	13.99	0.0002

Values are presented as mean ± SE.

**Table 2:** Estimates of the functional response parameters of *S. endius* to pupae of *B. dorsalis* and *M. domestica*.

Host Species	*α*	*T*_h_	*α*/*T*_h_	r^2^
*B. dorsalis*	0.6908 ± 0.5692	0.1458 ± 0.0657	4.7380	0.93
*M. domestica*	1.0399 ± 0.4275	0.0323 ± 0.0182	32.1950	0.97

Values are presented as mean ± SE. The values in parentheses represent 95% confidence intervals. *α*, attack rate; *T*_h_, handling time; *α*/*T*_h_, parasitism capacity.
